# Type 2 Myocardial Infarction in Young Adults: Insights From the National Readmission Database

**DOI:** 10.7759/cureus.19430

**Published:** 2021-11-10

**Authors:** Hadeer R Elsharnoby, Jaspreet Bhogal, Leonard Palatnic, Eman Elsheikh, Mahmoud Khalil, Waqas Kayani, Ahmed M Maraey

**Affiliations:** 1 Department of Physiology, Tanta University Faculty of Medicine, Tanta, EGY; 2 Internal Medicine, Midwestern University Arizona College of Osteopathic Medicine, Glendale, USA; 3 Department of Cardiology, Tanta University Faculty of Medicine, Tanta, EGY; 4 Internal Medicine, Lincoln Medical Center, New York City, USA; 5 Hospital Medicine, Catholic Health Initiatives St. Alexius Health, Bismarck, USA; 6 Family Medicine, University of North Dakota, Bismarck, USA; 7 Internal Medicine, University of North Dakota, Bismarck, USA

**Keywords:** predictors of re-admission, national readmission database., young adults, t2mi, type 2 myocardial infarction

## Abstract

Introduction

Type 2 myocardial infarction (T2MI) is an ischemic myocardial injury in the context of oxygen supply/demand mismatch in the absence of a primary coronary event. T2MI is often diagnosed in patients with a higher risk of morbidity and mortality. T2MI in young adults is poorly understood due to limited available data.

Methods

The Nationwide Readmission Database 2017-2018 was queried for admission with T2MI diagnosis in young adults (age ≤ 45 years). Index admissions with T2MI were identified. Other types of myocardial infarction and observations with missing data were excluded. December admissions were excluded to allow the 30-day follow-up. Cox proportional hazard multivariate regression model was used to determine predictors of readmissions. All P-values were two-sided, with 0.05 as the threshold for statistical significance.

Results

A total of 11,750 patients with a secondary diagnosis of T2MI were admitted between October 2017 and November 2018. The main primary etiologies of index admission were sepsis (14%) followed by hypertensive heart disease with heart failure (11%) and hypertensive emergency (7%), while main etiologies of readmission were hypertensive heart disease with heart failure (12%) followed by sepsis (9%) and acute kidney injury (3%). Valvular heart disease, chronic pulmonary disease, drug abuse, and depression were amongst the predictors of all-cause readmission.

Conclusion

We identified primary etiologies of admission and readmission, and predictors of readmissions in young adults presenting with T2MI. Further studies are needed to guide the management of T2MI in this age group.

## Introduction

Type 2 myocardial infarction (T2MI) is an ischemic myocardial injury in the context of oxygen supply/demand mismatch in the absence of a primary coronary event [1]. T2MI is often diagnosed in patients with a higher risk of morbidity and mortality [2]. T2MI is primarily thought to be a disease of the elderly. There has been increased recognition of T2MI in clinical practice amongst young adults. Nevertheless, T2MI in this age group is poorly understood due to limited available data.

## Materials and methods

This is a retrospective cohort study using the Agency for Healthcare Research and Quality’s Healthcare Cost and Utilization Project (HCUP) Nationwide Readmission Database (NRD) for year 2017-2018 [3]. The NRD is drawn from HCUP State Inpatient Databases (SID) containing verified patient linkage numbers that can be used to track a person across hospitals within a state while adhering to strict privacy guidelines. Unweighted, the NRD contains data from approximately 18 million discharges each year. Weighted, it estimates roughly 35 million discharges in the United States. The NRD contains both patient and hospital-level information. Up to 40 discharge diagnoses and 25 procedures are collected for each patient using International Classification of Diseases, Tenth Revision, Clinical Modification (ICD-10-CM) codes.

We included admissions between October 2017 and December 2018 with a secondary diagnosis of T2MI (ICD-10-CM code I21.A1). Analysis was restricted to this period as ICD-10-CM code for T2MI was introduced in October 2017. The T2MI ICD-10-CM code has been utilized in prior published research [4]. Patients were excluded if their age was less than 18 or more than 45 years, diagnosed with other types of myocardial infarction (types 1, 3, 4a, 4b, and 5), or had missing data. December discharges of each year were excluded to allow the 30-day follow-up.

The primary outcome was all-cause readmission within 30 days. A readmission was defined as any readmission within 30 days of the index admission. If the patient was readmitted multiple times during the 30 days post-admission, only the first readmission was included. Other objectives were to identify the primary etiology of index admission and readmissions.

Data analysis was performed using Stata 17 (StataCorp, College Station, TX). Data were expressed as percentages for categorical variables and means (standard deviations) for continuous variables. A Cox proportional hazard multivariate regression model was used to adjust for confounders and calculate the adjusted odds ratio (aOR) for readmission outcome. The model was built by including the variables that were associated with the outcome of interest on univariable regression analysis with a cut-off P-value of 0.20. All P-values were two-sided, with 0.05 as the threshold for statistical significance.

## Results

A total of 11,750 patients with a secondary diagnosis of T2MI were admitted between October 2017 and November 2018. The main primary etiologies of index admission were sepsis (14%) followed by hypertensive heart disease with heart failure (11%), hypertensive emergency (7%), heroin poisoning (2%), and pulmonary embolism (2%). The mean age was 36.6 years (±6.0). Males constituted most of our cohort (61.1%). Medicaid insurance was the primary insurance in 40.3% of patients. A total of 66.9% of our cohort had a median household income below the 50th percentile. Cardiac comorbidities were common, with heart failure (42.2%), complicated hypertension (41.7%), and cardiac arrhythmia (25.1%). A history of myocardial infarction or coronary revascularization was not common (5.1% and 3.4%, respectively). Drug abuse and tobacco abuse were present in 29.6% and 34.6%, respectively. Other baseline characteristics are presented in Table 1.

**Table 1:** Baseline characteristics of young adults diagnosed with type 2 myocardial infarction HIV: human immunodeficiency virus; AIDS: acquired immunodeficiency syndrome; PCI: percutaneous coronary intervention; CABG: coronary artery bypass graft

Baseline characteristics	Proportion
Mean age in years (SD)	36.6 ± 6.0
Female sex	38.9%
Insurance	
Medicare	18.3%
Medicaid	40.3%
Private	25.3%
Self	11.6%
Other	4.5%
Median household income quartile in percentile	
0-25th	38.8%
26th-50th	28.1%
51st-75th	21.3%
Above 76th	11.8%
Weekend admission	28.1%
Hospital bed size	
Small	12.1%
Moderate	24.4%
Large	63.6%
Teaching hospital	81.9%
Comorbidities	
Congestive heart failure	42.4%
Cardiac arrythmia	25.1%
Valvular heart disease	10.5%
Pulmonary circulation disorder	15.1%
Peripheral vascular disease	5.2%
Uncomplicated hypertension	16.5%
Complicated hypertension	41.7%
Chronic pulmonary disease	17.8%
Uncomplicated diabetes	10.0%
Complicated diabetes	17.5%
Hypothyroidism	5.9%
Chronic kidney disease ≥ Stage III	23.4%
Liver disease	15.5%
HIV/AIDS	1.4%
Connective tissue disease	4.5%
Coagulopathy	16.3%
Obesity	25.5%
Alcohol abuse	11.5%
Drug abuse	29.6%
Prior myocardial infarction	5.1%
Prior stroke	3.5%
Prior PCI	2.3%
Prior CABG	1.1%
Dyslipidemia	14.9%
Tobacco abuse	34.6%

From a total of 10,980 survivors, 2217 (20%) patients were readmitted within 30 days. Predictors of all-cause readmission were valvular heart disease (aOR: 1.24, 95% CI [1.02-1.49], P<0.01), chronic pulmonary disease (aOR: 1.24, 95% CI [1.07-1.42], P<0.01), liver disease (aOR: 1.37, 95% CI [1.15-1.62], P<0.01), solid tumor (aOR: 2.19, 95% CI [1.15-4.18], P=0.017), connective tissue disorder (aOR: 1.55, 95% CI [1.20-2.01], P<0.01), drug abuse (aOR: 1.21, 95% CI [1.04-1.41], P=0.014), depression (aOR: 1.21, 95% CI [1.03-1.41], P=0.017), chronic kidney disease ≥ Stage III (aOR: 1.41, 95% CI [1.11-1.77], P<0.01), and history of stroke or transient ischemic attack (aOR: 1.48, 95% CI [1.15-1.92], P<0.01).

The main primary etiologies of readmission were hypertensive heart disease with heart failure (12%) followed by sepsis (9%), acute kidney injury (3%), hypertensive emergency (2%), and acute hypoxic respiratory failure (2%). Type 1 myocardial infarction (T1MI) as a primary or secondary diagnosis (defined as ST-segment-elevation myocardial infarction [STEMI] or non-ST-segment-elevation myocardial infarction [NSTEMI]) was found in 138 (6%) of those readmitted within 30 days. Coronary angiography was performed in 112 patients, percutaneous coronary intervention in 18 patients, and coronary artery bypass grafting in 12 patients.

## Discussion

In our large retrospective analysis of 11,750 patients, we identified the primary etiologies of T2MI diagnosis in young patients ≤45 years old. We also identified predictors and etiologies of readmission in this age group. Amongst this age group, hypertensive heart disease with heart failure (12%) was the most common etiology of readmission. In contrast, it was the second most common etiology in index admissions for a T2MI (11%). Sepsis was the most common etiology for index admission of T2MI, but it was the second most common for readmissions (9%). When comparing these findings to the results found in the study by Stein et al., who examined patient characteristics, management, and outcomes of patients with T2MI, sepsis was found to be the second most common cause of T2MI in a cohort of 127 patients with a mean age of 75 ± 12 years [5]. This may lead to the notion that irrespective of age, sepsis remains a vital factor in both index admission and readmission. Notably, drug use, present in 29.6% of the patients, may be a risk factor for sepsis and therefore may explain why sepsis was found to be the second most common etiology of readmission in this cohort.

Moreover, heart failure was present in 42.4% of the patients in the age group experiencing an index admission with T2MI, and hence, it is expected to be a leading cause of readmission in this specific group of patients as it was responsible for 12% of the readmissions. This finding follows the trend for heart failure hospitalizations following a diagnosis of type 2 MI in patients of all age groups, as evidenced by the study by McCarthy et al. [6]. As such, similarly to sepsis, heart failure is a significant risk factor for both index admission and readmission, irrespective of age. Amongst this age group (<45 years), 38.6% of the patients admitted for a T2MI were female, compared to 43.4% in the study conducted by Stein et al. involving patients of all age groups [5]. In a study conducted by McCarthy et al. comparing sex discrepancies in T2MI, males were found to have more concurrent comorbidities when compared to their female counterparts, explaining the higher incidence of T2MI in the younger age group and the all-inclusive cohort. Readmission with T1MI occurred in 6% of all readmissions that emphasizes the increased risk of T1MI in this group.

Our study has certain limitations. Firstly, the database is administrative and uses ICD-10-CM codes for diagnoses that is subject to coding errors. Secondly, T2MI diagnosis may not be in accordance with the fourth definition of myocardial infarction. Thirdly, the retrospective analysis is subject to confounding bias despite rigorous adjustment.

## Conclusions

In conclusion, we identified primary etiologies of admission and readmission, and predictors of readmissions in young adults presenting with T2MI. Heart failure, complicated hypertension, and sepsis were common etiologies of T2MI in this age group. Further studies are needed to guide the management of T2MI in the younger population.
